# Engineering approaches for regeneration of T lymphopoiesis

**DOI:** 10.1186/s40824-016-0067-1

**Published:** 2016-06-29

**Authors:** Kyung-Ho Roh, Krishnendu Roy

**Affiliations:** The Wallace H. Coulter Department of Biomedical Engineering, Georgia Institute of Technology and Emory University, 950 Atlantic Drive NW, Atlanta, GA 30332 USA

**Keywords:** Thymus, T cells, Thymic epithelial cells, T cell receptor, Notch signaling, Negative selection, Positive selection, Stem cells, OP9-DL1

## Abstract

T cells play a central role in immune-homeostasis; specifically in the induction of antigen-specific adaptive immunity against pathogens and mutated self with immunological memory. The thymus is the unique organ where T cells are generated. In this review, first the complex structures and functions of various thymic microcompartments are briefly discussed to identify critical engineering targets for regeneration of thymic functions in vitro and in vivo. Then the biomimetic regenerative engineering approaches are reviewed in three categories: 1) reconstruction of 3-D thymic architecture, 2) cellular engineering, and 3) biomaterials-based artificial presentation of critical biomolecules. For each engineering approach, remaining challenges and clinical opportunities are also identified and discussed.

## Background

The thymus is the primary lymphoid organ that is uniquely responsible for T cell lymphopoiesis. It has long been established that the absence or malfunction of a thymus directly causes severe immunodeficiency due to the withdrawal of healthy peripheral T cells [1, 2]. Over the last few decades, much progress has been made in understanding the cellular and molecular details of how each thymic compartment collaborates to support the development of “healthy” T cell populations. The “healthy” T cells need to be able to distinguish “self” from “foreign” in a very specific, sensitive, and rapid manner to endow immunological protection from invading pathogens or malignant mutations. Considering this daunting task, various thymic compartments must be exceptionally well-orchestrated to support the development of functional T cells. Similar to other efforts in regenerative engineering of various complex organs, regeneration of thymic functions must be achieved based on the understanding of anatomy and functions of each thymic compartment. Therefore, our current understanding on the functional components of the thymus will first be discussed from the perspective of regenerative engineering. Then, recent engineering efforts to recapitulate each thymic compartment will be reviewed. From a clinical point of view, regenerative engineering of the thymus could be intended for multiple purposes including 1) support of in-vivo development of endogenous T cells, 2) robust generation of T cell precursors from readily available autologous or allo-matched donor stem cells, 3) induction of donor-specific immune tolerance to allografts [3], 4) in-vivo/in-vitro generation of antigen-specific T cells, and 5) simple in-vitro culture models to study T cell lymphopoiesis. Thus, we will discuss the engineering approaches of each thymic compartment along with the lines of thymic functions it could recreate, the relevant applications as well as the associated challenges.

## Review

1. Anatomy and functional compartments of the thymus
  The development of T cells (Fig. 1) starts by recruitment of bone-marrow-derived early T cell progenitor cells into the thymus. This initial cell population differentiates into serially distinctive stages while migrating through discrete compartments of the thymus until becoming mature T cells that return to the periphery. The collective knowledge on these anatomical regions regarding the sequential development of T cells in adult and embryonic thymus has been reviewed elsewhere [4, 5]. T cells in each developmental stage mature into the next step by interacting with the special cellular components in the region and migrate into the next region in concerted molecular signals given by chemokines, cytokines, adhesion molecules, lymphotoxins, and other developmental signals [6].

**Fig. 1:**
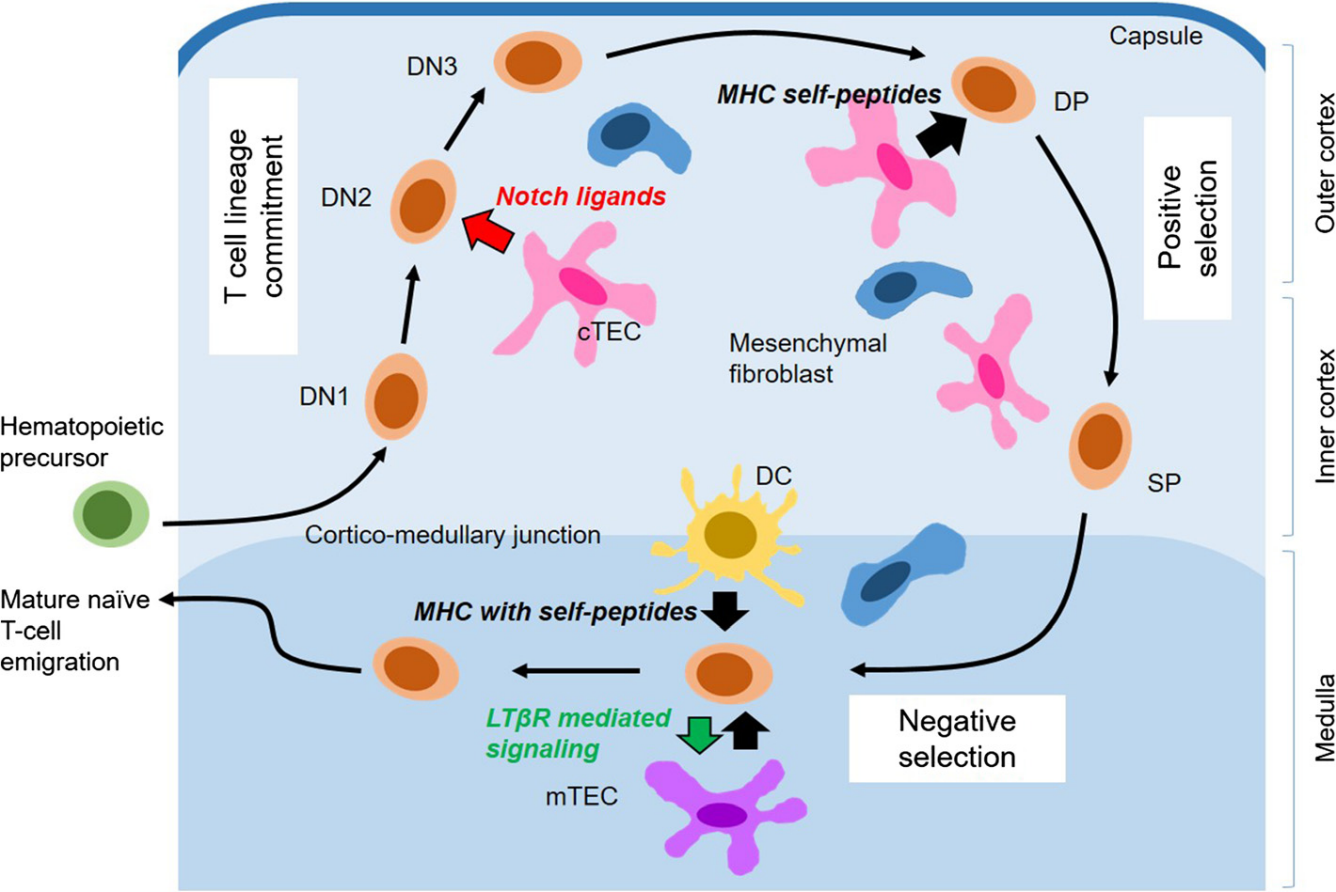
T cell development in thymic microcompartments. Bone-marrow derived hematopoietic stem/progenitor cells (green) enter the thymus through post-capillary venules and differentiate into T lineage cells (orange). Double negative (DN) thymocytes migrate outward in cortex (light blue region) as they interact with cortical TECs (cTECs, pink) and receive Notch signaling. DP thymocytes undergo positive selection as they migrate back to cortico-medullary junction interacting with pMHC expressed on cTECs. Positively selected thymocytes migrate into medulla (dark blue). SP thymocytes undergo the majority of negative selection within medulla by being tested for reactivity to tissue-restricted self-antigens expressed by medullary TECs (mTECs, purple) or dendritic cells (DCs, yellow). Mature SP thymocytes reciprocally promote maturation of mTECs by LTβR signaling. Mature T cells exit the thymus via blood or lymph. Modified from ref. [5] and [60]
  For regenerative engineering, it is beneficial to consider the thymic structure as an assembly line with serial modular compartments. First, a thymus is divided into two, the cortex and medulla. The early T cell progenitors enter the thymus near the cortico-medullary junction as CD4-CD8-double-negative (DN) cells. The DN stages are further subdivided based on the cell-surface expression of CD25 and CD44 (or CD117) [7]. The starting DN1 populations progress into DN2 and DN3 stages as they migrate outward, through the cortex, while interacting with cortical stromal cells, including thymic epithelial cells (cTECs) and mesenchymal fibroblasts. DN thymocytes further develop into CD4 + CD8+ double-positive (DP) cells, which migrate inward, back to the cortico-medullary junction, maintaining rich contacts with cTECs. Finally, mature CD4 + CD8- or CD4-CD8+ single-positive (SP) T cells migrate into the medulla, interact with medullary TECs (mTECs) and/or dendritic cells, until they finally egress the thymus into the peripheral blood.
  Throughout these developmental stages, the critical signals are provided by regional cellular and molecular components, which can be categorized into three types: 1) signals for T cell lineage commitment; 2) antigen recognition signals; and 3) cues for migration. These molecular signals are often closely related to each other with mutual causalities. First, the signals for lymphocyte stem/progenitor cells to develop into the T cell lineage are critical in the initial DN developmental steps, and produce universal effects in all thymocyte progenitor cells. However, the second signal that originates from antigen-recognition selectively affects the survival and developmental fate of a T cell clone with a unique T cell receptor (TCR). The special antigen recognition capacity of a T cell is conferred by the intricate affinity between TCR and peptide-major histocompatibility complex (pMHC). DP T cells actively survey the pMHC molecules expressed on the surface of cTECs. Only those T cells that can recognize the self-antigens receive survival signals and develop further (positive selection). But once these positively-selected T cells reach the medulla as SP T cells, they will interact with mTECs and DCs, and the T cells that recognize tissue-specific self-antigens with “higher” affinities undergo apoptosis (negative selection) to prevent autoimmunity. Lastly, there are concerted molecular mechanisms that allow developing T cells to migrate between regional compartments, one after another, which involves various chemokines and adhesion molecules. Excellent reviews that further detail the molecular interactions and developmental signaling events can be found elsewhere [8, 9]. It should be noted that the cellular communications between developing T cells and thymic stroma are reciprocal, i.e. while developing T cells receive above-mentioned complex molecular signals, they also provide critical signals to the thymic stromal cells for their survival and function [10, 11].
  Even though the majority of previous studies on T cell development were performed on αβ T cells, namely either CD4+ helper T cells or CD8+ cytotoxic T cells, knowledge on the ability of thymic microenvironments to support the development of other thymocyte lineages, such as NKT cells [12–14], γδ T cells [15–20], and Foxp3 + CD4 + CD25+ regulatory T (T_reg_) cells [21–23] is also accumulating. All of these studies likewise provide significant insights on engineering those special niches for regenerative purposes.
2. Engineering for regeneration of thymic functions
  In order to recapitulate the unique structures and functions of the thymus and its compartments, efforts have been made in three directions: 1) reconstruction of 3-dimensional (3-D) thymic stromal cells (TSCs) network, 2) cellular engineering, and 3) artificial presentation of developmental signals (Table 1).

**Table 1:** Characteristic features, applications, advantages, and limitations of engineering approaches for T lymphopoesis

Engineering approaches	Applications	Advantages (+) and limitations (−)	Representative and notable references
1) Reconstruction of TSC’s 3-D network			
• Fetal thymic organ culture (FTOC) • Reaggregate thymus organ culture (RTOC) • Artificial scaffolds • Decellularized thymic scaffolds	• To study T cell tolerance and MHC restriction in vitro • To study thymopoiesis in vivo upon grafting into an ectopic locations of athymic mice	• Simple and straightforward design (+) • Absolute dependency on biopsy and isolation of thymus or thymic cells (−) • Limited culture sizes of 3-D platforms (−) • Limited number of T cells that can be generated in vitro (−)	• FTOC [27, 31] • RTOC [38] • Grafting in ectopic locations [32, 36] • Artificial scaffolds [40, 41, 44] • Decellularized scaffolds [45]
2) Cellular Engineering			
• Differentiation of stem cells into TSCs • Genetic introduction of effector molecules that define TSC functions to cell lines or somatic cells • Cell reprogramming	• To use human pluripotent stem cells for regeneration of thymus or induction of immune tolerance • To generate T cell precursors and functional T cells using robust 2-D culture platforms in vitro	• Use of clinically relevant, endogenous stem cell sources (+) • Use of readily available 2-D culture platforms for recapitulation of T lymphopoesis in vitro (+) • Potential xenogenic cross-contamination (OP9-DL1) (−) • Ineffective positive selection of CD4+ T cells (OP9-DL1) (−) • Need for complex genetic modifications and related risk of viral contamination (−)	• mESC to TEPC [50, 51] • hESC to TEC [53, 54] • OP9-DL1 [59] • Clinical usage of OP9-DL1 platform [67–69] • Cell reprogramming [49]
3) Biomaterials-driven artificial presentation of developmental signaling molecules			
• Plate- or bead-bound Notch ligands for differentiation of T precursors from various stem cells • Use of pMHC tetramer to induce antigen specificity on developing T cells	• To generate T precursors from various stem cells in vitro, which later can be employed in adoptive cell therapies. • To induce or selectively expand antigen-specific T cells	• Potential realization of purely biomaterial-based T lymphopoesis ex vivo (+) • Requirement for expensive recombinant proteins (−) • Generation of potentially self-reactive T cells due to lack of negative selection (−) • Limited T cell expansion (−)	• Notch ligands [74, 75, 77, 79] • pMHC tetramer [80, 81]

  - Reconstruction of 3-D network architecture of TSCs
    Unlike B-cell development, which can be recapitulated in vitro by co-culture of hematopoietic stem/progenitor cells on a 2-dimensional (2-D) layer of bone-marrow-derived stromal cell lines [24], T cell development cannot be successfully reproduced by using TECs cultured on 2-D environments. Instead, TECs cultured on a 2-D environment lose the expression of characteristic genes [25], and differentiate into skin cells or express markers of other terminally differentiated epithelial cells [26]. Naturally, TECs in the thymus form a very unique, sponge-like 3-D network structure [11], which might be critical for rich crosstalk between developing T cells and thymic stromal cells as well as migration of developing T cells.
    Even before the full molecular details of how 2-D vs. 3-D environments affect the gene expression of TECs were elucidated, fetal thymic organ culture (FTOC) was introduced as a simple method to culture TECs in 3-D and achieve in-vitro T cell development [27–30]. FTOC has served as the main in-vitro culture platform to study T-cell development, and various modifications have also been introduced. For example, Jenkinson et al. made use of deoxyguanosine, which is selectively toxic to mature T cells, to deplete the endogenous T cells from FTOC while maintaining the surviving stromal elements [31]. The “emptied” FTOC can be recolonized by stromal and lymphoid cells of different haplotypes, and has become a great platform to study T cell tolerance and MHC restriction in vitro. Meanwhile, grafting fetal thymus tissue in an ectopic location of an immune-deficient murine model has provided means to study thymopoiesis in vivo. The most established example is the grafting of fetal thymus tissue under the kidney capsule [32]. The neovascularization within the grafted tissue allows for continuous entry of T cell progenitors and further development of the grafted tissue to an ectopic, functional thymus. It was also shown that co-implantation of human fetal liver and human fetal thymus under the kidney capsule could establish humanized mouse models [33] to study human T lymphopoiesis with or without concurrent intravenous administration of human hematopoietic stem progenitor cells [34, 35]. Ectopic grafts of murine fetal thymus lobes were also achieved by a tissue engineering methodology named the mouse chamber (MC) model [36]. In this model, murine thymus lobes and extracellular matrix containing growth factors are enclosed together within a silicone tube which is then implanted around the epigastric vessels of the groin vascular bed. The murine fetal thymus tissue was successfully engrafted and vascularized in the chamber, and supported de novo generation of functional T cells [36].
    Instead of using an intact fetal thymus tissue, a single cell suspension of thymic stroma can be prepared by enzymatic degradation of deoxyguanosine-treated fetal thymic lobes. When thymic stromal cells are reaggregated together with HSC-derived T progenitor cells and cultured in a hanging drop or on culture sponge (named as reaggregate thymus organ culture, RTOC), it also supports T cell development in vitro [37, 38]. More recently, Chung et al. reported that EPCAM + CD45-CD105- TECs and CD45-EPCAM + CD105+ thymic mesenchyme (TM)-enriched populations can be separately isolated from postnatal human thymus using specific culture conditions, and used for RTOC with human cord blood-derived CD34+ hematopoietic stem and progenitor cells (HSPCs) [39]. This aggregate culture supported T cell generation to a certain degree in vitro as well as in vivo following inguinal implantation in immune deficient mice. When TM engineered to express vascular endothelial growth factor via lentiviral transfection was employed, the implanted reaggregates showed enhancement in size and T cell production.
    3-D culture of thymic stromal cells was also enabled by using artificially engineered scaffolds. A confluent layer of murine thymic stroma was generated on top of tantalum-coated reticulated structure of carbon matrices, which showed syngeneic or xenogeneic support for generation of functional murine [40] or human [41] T cells in vitro upon co-culture of thymus- or bone marrow-derived hematopoietic progenitor cells.
    Among the various functional supports provided by each cellular component in the thymus, mTECs are responsible for expressing a host of tissue-restricted antigens, termed “promiscuous gene expression”, which is critical for the negative selection of self-reacting T cells [42]. However, expression of the critical molecular components for mTECs functional features, such as Aire and transcription factor forkhead box N1 (FoxN1), are known to be downregulated upon 2-D culture of TECs [26, 43]. Motivated by phenotypic similarities between mTECs and keratinocytes, Pinto et al. cultured mTECs on top of a 3-D co-culture system that previously had shown in-vitro skin development [44]. mTECs proliferated and sustained the expression of Aire and FoxN1 when they were cultured on a 3-D non-woven fibrous scaffold seeded with skin-derived dermal fibroblasts in fibrin gel. Even though this culture system showed a new possibility to recapitulate promiscuous gene expression by mTECs in vitro, an emulation of functional negative selection in de novo T cell regeneration was not attempted.
    Recently, a more robust reconstruction of thymus organoids was achieved using decellularized thymic scaffolds [45]. The acellular scaffold can be prepared by a detergent-perfusion based method that enables the clearance of cellular components while retaining the original 3-D architecture and extracellular matrix (ECM) of various organs [46–48]. Upon repopulating the 3-D acellular thymic scaffold with thymic stromal cells (TSCs) and BM derived Lin- progenitor cells, various components of TSCs including TECs, endothelial cells, and thymic fibroblasts, remained viable for more than 3 weeks, and supported in vitro T cell generation. When transplanted into athymic nude mice, these thymus organoids effectively function to support induction of T cell-dependent humoral and cellular immunity. Moreover, induction of tolerance to allograft was achieved by employing thymus organoid constructed with mixture of TECs derived from the donor and the recipient mice.
    Engineering approaches mentioned above have proven to be versatile and useful in fundamental research as well as potential clinical applications in vitro and in vivo. However, the universal need for isolation of thymic stroma from the patient and the limited size and number of cells generated by in-vitro methods make it difficult to apply these methods more widely.
  - Cellular Engineering Approaches
    For robust regeneration of thymic functions, each cellular component is an interesting target for cellular engineering. Historically, two lines of activity have yielded prominent progresses: 1) differentiation of stem cells into various thymic stromal cells, 2) genetic introduction of key molecules that define thymic functions [6] or transcription factors [49] to cell lines or other somatic cells. Both approaches could potentially negate the absolute dependency on TSCs isolated from thymus biopsies and/or need of complex 3-D culture condition.
    Mouse embryonic stem cells (mESC) were successfully differentiated to thymic epithelial progenitor cells (TEPCs), which could further differentiate to both cortical and medullary TECs [50]. When placed in vivo, these mESC-derived TECs could support thymopoiesis upon either syngeneic [50] or allogenic [51] bone marrow transplant. Moreover, mESC-derived TEC-treated mice developed tolerance against host and mESCs upon allo-BM transplant [51]. Using a separate method, Inami et al. successfully differentiated induced pluripotent stem cells (iPSCs) to TEPC and mTEC phenotypes [52]. More recently, functional TECs were also generated from human embryonic stem cells (hESCs) by precise regulation of TGFb, BMP4, RA, Wnt, Shh, and FGF signaling [53], or by sequential regulation of Activin, retinoic acid, BMP, and WNT signals [54]. These studies made important progress towards employing human pluripotent stem cells for thymus transplantation, particularly for clinical purposes such as induction of tolerance to stem-cell-derived cell-therapeutics, or restoration of senescing thymic functions.
    As mentioned above, 3-D culture condition had been regarded as mandatory for TEC to support T cell development in vitro. In comparison, early hematopoiesis and B cell lymphopoiesis can be well supported by a monolayer (2-D) culture of bone-marrow-derived stromal cell line such as OP9 [55, 56]. This bone marrow stromal cell line was derived from OP/OP mice that are deficient in macrophage colony-stimulating factor (MCSF), which in turn contributed to the ability of OP9 cells to preferentially support differentiation of HSCs to B cells over myeloid cells. Inspired by the two separate findings that 1) Notch signaling plays important roles at various stages of T cell development [57, 58], and 2) OP9 cells constitutively express both members of jagged-gene family but fail to support T cell generation, OP9 cells were engineered to express another Notch ligand, delta-like 1 (OP9-DL1) by retroviral transduction [59]. Indeed, the 2-D monolayer of OP9-DL1 cells could support T-cell differentiation from HSCs, including the generation of DP and CD8+ SP cells [59, 60], thus changing the paradigm of 3-D requirements for in-vitro T lymphopoiesis. Later, it was proven that the TSCs cultured in 2-D monolayer indeed lose the expression of Notch ligands, delta-like ligand (DLL)1 and DLL4, which has a direct causal effect on the loss of ability to support T lymphopoiesis [61]. It is noteworthy that ectopic re-expression of Notch ligands was sufficient for the 2-D cultured TSCs to support T cell generation.
    Despite the robust induction of functional T cells from multiple sources of stem cells [62–65], OP9-DL1 co-culture has some limitations. One of the main drawbacks is the lack of MHC class II or CD1d expression in OP9 cells, which consequently hampers their support for the positive selection of CD4+ T cells and NKT cells, respectively [60]. In addition, OP9 cells do not express a host of tissue-restricted antigens like mTECs, thus proper negative selection of the self-reactive TCR repertoire is lacking. Nevertheless, functionally mature T cells including CD4+ and CD8+ were obtained from HSCs by OP9-DL1 co-culture systems, which indicate that some level of positive selection is induced by interaction between T precursor cells [66].
    A couple of exciting strategies were developed to utilize the OP9-DL1 system for clinical applications by avoiding the need of rigorous selection processes in vitro. First, OP9-DL1 co-culture system was employed to robustly generate T cell precursors from HSCs. The generated T precursors were adoptively transferred to lethally-irradiated allogeneic recipient mice together upon bone-marrow transplantation. Adoptively transferred T-cell precursors could give rise to enhanced T-cell dependent immunity against infection and significant graft-versus-tumor (GVT) activity but without graft-versus-host disease (GVHD) [67]. Second, two separate studies have utilized OP9-DL1 for generation of cytotoxic T cells from human HSCs genetically modified to express tumor- or virus- specific TCRs, which negated the need of TCR selection processes [68, 69].
    A human TEC line expressing human leukocyte antigens (HLAs) was genetically engineered to overexpress murine DLL1 as an alternative strategy to utilize both Notch signaling and interactions between TCRs and pMHC molecules [70]. This TEC-DL1 line promoted generation of DP T cells from BM- and CB-derived hematopoietic precursor cells (HPCs). However, how the presence of HLAs contributed in this de novo generation of T-lineage committed cells (such as positive selection) still remains to be studied.
    Instead of genetically introducing individual key effector molecules that define thymic functions, Bredenkamp et al. reprogrammed primary mouse embryonic fibroblasts into functional TECs by forced expression of the transcription factor FoxN1, which is critically required for development of TECs [49]. On a regular 2-D culture platform, FoxN1-induced TECs supported efficient development of CD8+ as well as CD4+ T cells in vitro, owing to their capacity to express both MHC class I and II. When the cellular aggregates of these FoxN1-induced TECs, thymocytes, and TM cells are grafted under the kidney capsule, they develop into spatially organized cortex and medulla of a fully functional thymus unit. Despite some practical hurdles that prevent direct application of a similar technology to human patients, this cell reprogramming method shows tremendous potential towards patient-specific custom-designed T cell therapies.
    In terms of clinical applications, neither using patient derived TSCs nor employing OP9-DL1 cells are perfectly ideal for the derivation of T cells from stem cells. Thymic biopsy or an equivalently invasive procedure is required to acquire TSCs, and the number of TSCs that you can get is normally limited. Using xenogenic materials such as OP9 cells is not ideal due to potential cross-contamination, not to mention other limitations considered above. Clark et al. reported that functional human T cells can be induced from HPCs by co-culturing them on a tantalum-coated reticulated carbon matrix seeded with human-skin derived fibroblasts and keratinocytes, replacing conventionally employed TSCs and TECs, respectively [71]. Moreover, de-novo generated T cells possessed a diverse TCR repertoire, and were functionally mature but tolerant to self-MHC; this strongly indicates successful performance of positive and negative selection processes in vitro. However, the reproducibility of some of these exciting findings have been questioned by others [72], so a certain reconciliation within the field is awaiting.
  - Biomaterials-driven artificial presentation of signaling molecules
    As shown above, Notch signaling provided by Notch ligands, DLL1 and DLL4, has been identified as the critical molecular components for induction of T cell-lineage commitment from stem cells [73]. Therefore, there is a potential to recapitulate effective T lymphopoiesis by induction of proper Notch signaling using artificial presentation of Notch ligands.
    Towards this goal, some very important studies have provided background knowledge and engineering strategy. First, it was shown that the extracellular domain of Notch ligands need to be immobilized onto a surface to effectively induce Notch signaling, while soluble counterparts bind to Notch receptor without signaling induction, thus they are inhibitory to Notch function [74]. Relatively low surface densities of Delta-1 support differentiation of early T cell precursors as well as B cell precursors, while higher densities of Delta-1 suppress B cell-lineage commitment [75]. The two Notch ligands, DLL1 and DLL4, both of which are critical for development of T cell lineage, perform differently in terms of inhibition of B and myeloid-specific transcription factors at equivalent lower density limits [73]. In broader sense, the surface density of Notch ligands becomes an important determinant in regulating hematopoietic cell-fate outcomes, affecting ex-vivo differentiation as well as in-vivo marrow repopulating ability of cord blood [76].
    It was shown that plate-bound Delta-1 could expand and induce T cell precursors from either human CD34 + CD38- cord blood cells [77] or lin-Sca-1 + c-kit + (LSK) murine HSCs [78], which resulted in enhanced T cell thymic reconstitution upon adoptive transfer of these cells. Instead of plate-bound form and a defined media, Taqvi et al. utilized DLL4-functionalized microbeads and insert co-culture on top of an OP9 cell monolayer to successfully generate Thy1.2+ early T cells from LSK HSCs without direct OP9 stromal cell contact [79].
    It is noteworthy that in these culture conditions with artificially presented Notch ligands, proper cytokine mixture needs to be supplemented either by defined composition of recombinant components or by conditioned media obtained by stromal cell culture. And despite the successful generation of early T cells mentioned above, mature and functional SP T cells have hardly been achieved by these biomaterial-based in-vitro culture systems.
    The other critical molecular components provided by thymic stroma to developing T cells are pMHC molecules. Lin et al. demonstrated that both thymus-derived DP thymocytes and murine ESC-derived cells can be differentiated into CD8+ SP T cells by using MHC tetramers during differentiation culture. The resulting CD8+ T cells showed significant cytotoxic T lymphocytes activity against target cells loaded with the same antigen as used in the culture [80]. More recently, a similar strategy was also applied to human umbilical cord blood derived CD34 + CD38-/low human HSCs [81]. CD1a + CD7+ and DP T cells were differentiated from human HSCs using plate-bound DLL1, and subsequently cultured with tetramers of HLA-A*0201 restricted cytomegalovirus (CMV) or influenza epitopes. Again, the resulting CD8+ SP T cells cultured with each antigen showed in-vitro cytolytic functionality against corresponding peptide-loaded target cells in an antigen-specific manner [81]. Even though such in-vitro generation of functional, antigen-specific T cells from clinically relevant cell sources could eventually provide a new opportunity in adoptive transfer immunotherapies, there remain some important questions: How specific are the new TCRs? Are these new TCRs self-reactive? Can these antigen-specific T cells be significantly expanded in number? Efforts to answer to these questions and further engineering strategies to properly incorporate positive and negative selection processes into in-vitro T lymphopoiesis would warrant a safer, more versatile and robust clinical applicability.

## Conclusions

There are great and growing needs for engineering approaches to regenerate T lymphopoieis in vitro as well as in vivo. In many developed countries, the demographic structure is getting older and reverting age-related thymic atrophy is becoming more important. In addition to providing means to cure the thymic atrophy caused by diseases such as DiGeorge syndrome [82], severe combined immunodeficiency syndrome (SCID) [83] and various infections [84, 85], de-novo T cell generation from various stem and progenitor cells has a great potential in T cell adoptive transfer immunotherapy for numerous cancers, which recently attracted huge interests from academia as well as the pharmaceutical industry [86–88]. In order to translate its immense clinical promise and rapid scientific progress into clinics, adoptive T cell transfer therapy needs to find scalable technologies for providing functional T cells from HLA-matched, readily available sources. As discussed in this review, each engineering approach for T cell generation has its own advantages and disadvantages (Table 1), and must find its most appropriate use in diverse applications. Nevertheless, development of more robust engineering approaches to recapitulate full arrays of thymic functions, namely, T lineage commitment, and rigorous positive/negative selections in a clinically scalable fashion is an ongoing task.

## Abbreviations

BM, bone marrow; CB, cord blood; cTEC, cortical thymic epithelial cell; DC, dendritic cell; DL, delta-like; DLL, delta-like ligand; DN, double negative; DP, double positive; ECM, extracellular matrix; FTOC, fetal thymic organ culture; HLA, human leukocyte antigen; HSC, hematopoietic stem cell; HSPC, hematopoietic stem and progenitor cell; iPSC, induced pluripotent stem cell; mESC, mouse embryonic stem cell; mTEC, medullary thymic epithelial cell; NKT, natural killer T; pMHC, peptide major histocompatibility complex; RTOC, reaggregate thymus organ culture; SP, single positive; TCR, T cell receptor; TEC, thymic epithelial cell; TEPC, thymic epithelial progenitor cell; TM, thymic mesenchyme; TSC, thymic stromal cell
